# Risk factors for adverse clinical outcomes with COVID-19 in China: a multicenter, retrospective, observational study

**DOI:** 10.7150/thno.46833

**Published:** 2020-05-15

**Authors:** Peng Peng Xu, Rong Hua Tian, Song Luo, Zi Yue Zu, Bin Fan, Xi Ming Wang, Kai Xu, Jiang Tao Wang, Juan Zhu, Ji Chan Shi, Feng Chen, Bing Wan, Zhi Han Yan, Rong Pin Wang, Wen Chen, Wen Hui Fan, Can Zhang, Meng Jie Lu, Zhi Yuan Sun, Chang Sheng Zhou, Li Na Zhang, Fei Xia, Li Qi, Wei Zhang, Jing Zhong, Xiao Xue Liu, Qi Rui Zhang, Guang Ming Lu, Long Jiang Zhang

**Affiliations:** 1 Department of Medical Imaging, Jinling Hospital, Medical School of Nanjing University, Nanjing, Jiangsu, 210002, China.; 2 Department of Medical Imaging, Xiaogan Central Hospital of Wuhan University of Science and Technology, Xiaogan, Hubei, China.; 3 Huanggang Central Hospital, No. 11, Kaopeng Road, Huangzhou District, Huanggang, Hubei, China.; 4 Department of Medical Imaging, Shandong Provincial Hospital Affiliated to Shandong First Medical University, Jinan, Shandong, China.; 5 Department of Radiology, the Affiliated Hospital of Xuzhou Medical University, Xuzhou, Jiangsu, China.; 6 Institute of Medical Imaging and Digital Medicine, Xuzhou Medical University, Xuzhou, Jiangsu, China.; 7 Department of Radiology, Xiangyang Central Hospital, Affiliated Hospital of Hubei University of Arts and Science, Xiangyang, Hubei, China.; 8 Department of Medical Imaging, the Affiliated Anqing Hospital of Anhui Medical University, Anqing, Anhui, China.; 9 Departments of Infectious Disease & Department of Medical Imaging, Wenzhou Central Hospital, Wenzhou, Zhejiang, China.; 10 Department of Radiology, Hainan General Hospital, Haikou, Hainan, China.; 11 Department of Medical Imaging, Jingzhou Central Hospital, The Second Clinical Medical College, Yangtze University, Jingzhou, Hubei, China.; 12 Department of Medical Imaging, The Second Affiliated Hospital and Yuying Children's Hospital of Wenzhou Medical University, Wenzhou, Zhejiang, PR China.; 13 Department of Medical Imaging, Guizhou Provincial People's Hospital, Guiyang, China.; 14 Department of Medical Imaging, Taihe Hospital, Hubei University of Medicine, Shiyan, Hubei, China.; 15 Department of Radiology, General Hospital of the Yangtze River Shipping, Wuhan, Hubei, China.; 16 Department of Radiology, Yichang Central People's Hospital, Yichang, Hubei, China.; 17 Department of Radiology, The First Hospital of China Medical University, Shenyang, Liaoning, China.

**Keywords:** COVID-19, Coronavirus, Pneumonia, Risk factor, Mortality

## Abstract

**Background:** The risk factors for adverse events of Coronavirus Disease-19 (COVID-19) have not been well described. We aimed to explore the predictive value of clinical, laboratory and CT imaging characteristics on admission for short-term outcomes of COVID-19 patients.

**Methods:** This multicenter, retrospective, observation study enrolled 703 laboratory-confirmed COVID-19 patients admitted to 16 tertiary hospitals from 8 provinces in China between January 10, 2020 and March 13, 2020. Demographic, clinical, laboratory data, CT imaging findings on admission and clinical outcomes were collected and compared. The primary endpoint was in-hospital death, the secondary endpoints were composite clinical adverse outcomes including in-hospital death, admission to intensive care unit (ICU) and requiring invasive mechanical ventilation support (IMV). Multivariable Cox regression, Kaplan-Meier plots and log-rank test were used to explore risk factors related to in-hospital death and in-hospital adverse outcomes.

**Results:** Of 703 patients, 55 (8%) developed adverse outcomes (including 33 deceased), 648 (92%) discharged without any adverse outcome. Multivariable regression analysis showed risk factors associated with in-hospital death included ≥ 2 comorbidities (hazard ratio [HR], 6.734; 95% CI; 3.239-14.003, p < 0.001), leukocytosis (HR, 9.639; 95% CI, 4.572-20.321, p < 0.001), lymphopenia (HR, 4.579; 95% CI, 1.334-15.715, p = 0.016) and CT severity score > 14 (HR, 2.915; 95% CI, 1.376-6.177, p = 0.005) on admission, while older age (HR, 2.231; 95% CI, 1.124-4.427, p = 0.022), ≥ 2 comorbidities (HR, 4.778; 95% CI; 2.451-9.315, p < 0.001), leukocytosis (HR, 6.349; 95% CI; 3.330-12.108, p < 0.001), lymphopenia (HR, 3.014; 95% CI; 1.356-6.697, p = 0.007) and CT severity score > 14 (HR, 1.946; 95% CI; 1.095-3.459, p = 0.023) were associated with increased odds of composite adverse outcomes.

**Conclusion:** The risk factors of older age, multiple comorbidities, leukocytosis, lymphopenia and higher CT severity score could help clinicians identify patients with potential adverse events.

## Introduction

Coronavirus Disease-19 (COVID-19) was initially reported in Wuhan, Hubei Province, China, in December, 2019 and rapidly spread to all other provinces of China and throughout the world 1-2. Despite the absence of targeted antiviral drugs and vaccines, the outbreak in China was preliminary contained by means of symptoms surveillance and patient isolation 3. As of May, 3, 2020, there have been 84,393 confirmed cases of COVID-19 in China 4. However, the situation abroad was not optimistic. On March 11, 2020, the World Health Organization (WHO) declared the outbreak as a pandemic and stated that Europe had become the epicenter of the pandemic 5. As of May, 4, 2020, there were 3,351,494 confirmed cases outside China, and a total of 239,604 patients lost their lives in this disaster 4, which has raised wider public concern. The coronavirus pandemic is a serious crisis in history and a timely and effective summary of the Chinese data will be of considerable value for individuals who are at risk.

Although evidences related to the death and adverse outcomes of COVID-19 are rapidly accumulating, most studies focused on the comparison of clinical characteristics between deceased and recovered patients 6-8. Some researchers have revealed prognosis information; however, the data were mainly from Wuhan, limited by relatively small sample sizes, single-center observations, using univariable analysis alone or lack of clear clinical outcomes for all patients 9-13, which cannot represent the overall situation in China. In this study, we systematically analyzed the clinical, laboratory and CT imaging data of laboratory confirmed COVID-19 patients with clear prognostic information in 16 tertiary hospitals from 8 provinces of China and identified the risk factors associated with in-hospital death as well as adverse outcomes. We believe that the baseline data associated with death and adverse outcome will be of considerable value for individuals who are most likely to benefit from timely intensive care.

## Methods

### Study Design and Participants

**Figure 1:**
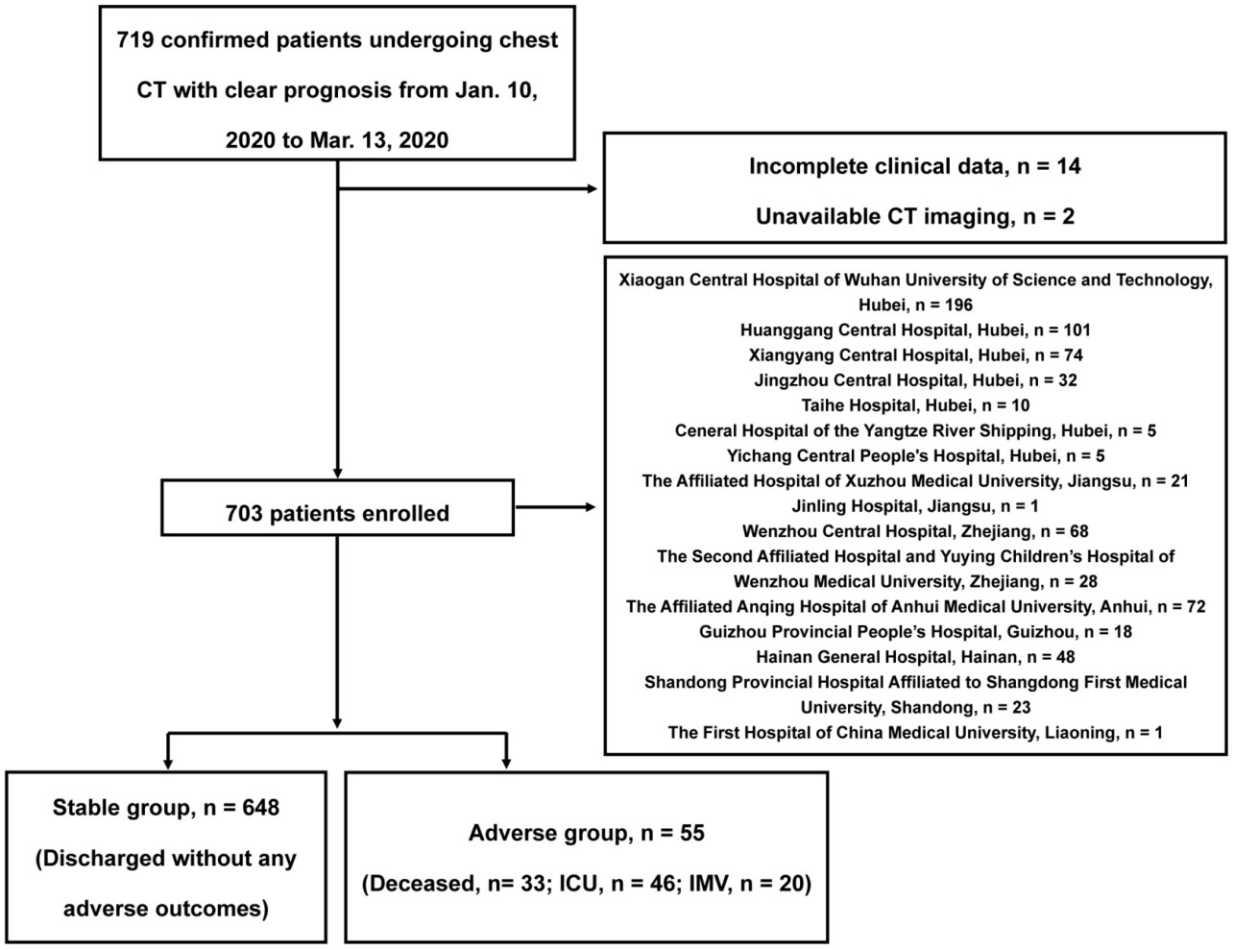
** Flowchart of This Study.** ICU: intensive care unit; IMV: invasive mechanical ventilation support.

In this retrospective observational study, our data were from 8 provinces, including 5 of the top 10 provinces with the number of the most confirmed cases in China, that is, Hubei Province, Zhejiang Province, Anhui Province, Shandong Province, and Jiangsu Province, see **Figure S1** for distribution. Patients admitted from January 10, 2020 to March 13, 2020 were preliminarily included according to the criteria as following: (a) severe acute respiratory syndrome coronavirus-2 (SARS-CoV-2) was confirmed by reverse-transcription-polymerase-chain-reaction (RT-PCR); (b) thin-section chest CT scan was performed; (c) clear prognosis information was available (discharge, or adverse outcomes including in-hospital death, the admission to intensive care unit [ICU] and requiring invasive mechanical ventilation support [IMV]). After excluding 14 patients with incomplete clinical data and 2 patients without available CT images, a total of 703 in-patients were finally included, the study flowchart is shown in **Figure 1**. This study protocol was approved by the institutional review board of Jinling Hospital, Medical School of Nanjing University (2020NZKY-005-02), written informed consents were waived.

### Data Collection

We reviewed clinical electronic medical records, laboratory results, and radiological findings of all included COVID-19 patients. The following clinical data were collected: age, sex, occupation, exposure history, onset symptoms (fever, cough, myalgia, fatigue, headache, nausea, abdominal pain, diarrhea, dyspnea, no symptom), signs, and comorbidity (history of cardiovascular disease, diabetes, hypertension, chronic obstructive pulmonary disease [COPD], chronic liver disease, chronic kidney disease, malignancy). According to the clinical manifestations based on the Diagnosis and Treatment Program of 2019 New Coronavirus Pneumonia (trial sixth version), confirmed patients were divided into mild, moderate, severe, and critical types 14,15. Laboratory results included complete blood counts, clotting profiles, biochemical tests (liver and kidney function, electrolytes, creatine kinase and lactate dehydrogenase). All patients underwent chest CT scans and the frequency of CT examinations was determined by the attending physicians. All data were provided by each center and checked by two physicians (S.L. and P.P.X.). Missing data needed to be reconfirmed and clarified, we kept in touch with all centers.

### CT Image Acquisition and Interpretation

**Figure 2:**
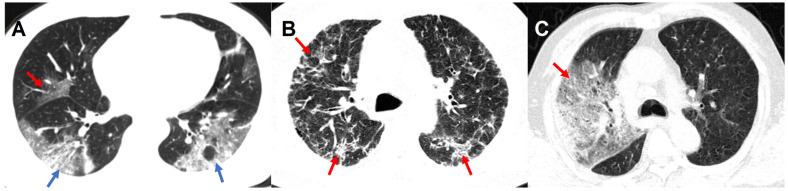
** Representative CT Images of confirmed COVID-19 pneumonia. Panel A:** A 46-year-old male patient presented with fever, cough, and fatigue without a clear exposure history. Irregular GGO (red arrow) can be seen in the middle lobe of the right lung, and large patchy irregular GGO with consolidation (blue arrow) can be seen in the lower lobes of both lungs. **Panel B:** A 56-year old male patients presented with fever, cough, fatigue and headache without a clear exposure history. Extensive diffuse pneumonia can be seen in both lungs with multiple ILD (red arrow). **Panel C:** A 62-year-old male patients presented with fever, cough, myalgia as well as diarrhea, and had a close contact with confirmed COVID-19 patients. In the context of GGO with consolidation, crazy-paving pattern (red arrow) can be seen in the right lung. COVID-19: Coronavirus Disease-19; GGO: ground-glass opacity; ILD: interstitial lung disease.

Chest CT scans were routinely performed using ≥ 16 slice multidetector CT scanners without use of contrast agents. Scanners and scanning protocols in details can be found in **Supplementary materials**. All Digital Imaging and Communications in Medicine (DICOM) images were reviewed by four radiologists (Z.Y.S, L.Q, F. X. and J. Z.) with 18, 6, 5 and 5 years of experiences in thoracic radiology in core lab in Jinling Hospital, Medical School of Nanjing University, respectively. They independently evaluated initial CT images without access to patient's clinical or laboratory results. The following CT features were analyzed and recorded: lesion distribution, morphology and number of lesions; main signs including pure ground-glass opacity (GGO), pure consolidation, GGO with consolidation, interstitial lung disease (ILD), crazy-paving pattern and other abnormalities. The number of affected lung lobes and lung segments were recorded (**Supplementary Materials**). We also proposed a lung segment-based CT severity score to assess the severity of the pneumonia. Some representative CT signs are shown in** Figure 2** and more detailed CT image features are summarized in **Supplementary Materials**. Any disagreement was resolved through discussions and consultations.

### Outcomes

The primary outcome was in-hospital death and the secondary outcomes were composite clinical adverse outcomes including in-hospital death, admission to ICU and requiring IMV. Discharge criteria included: no fever for at least 3 days, significant improvement on chest CT in both lungs, clinical relief of respiratory symptoms, and repeated negative RT-PCR results at ≥ 24 hours interval 9. The time interval between the onset of symptoms and the development of adverse outcomes or discharge were recorded.

### Statistical Analysis

All analyses were conducted using SPSS (version 22.0, IBM SPSS Inc., Chicago, IL, USA), Medcalc (version 11.0, MedCalc Software, Ostend, Belgium), R software (version 3.1.1, R Foundation for Statistical Computing) and PASS (version 11.0, NCSS, Kaysville, USA). Kolmogorov-Smirnov test was used to evaluate the normality of quantitative data. Mean and standard deviation (SD) were used to describe normally distributed data, while median and interquartile range (IQR) was used to describe non-normally distributed data. Categorical variables were presented as numbers and percentages. Mann-Whitney U test, Pearson χ^2^ test and Fisher's exact test were used to compare variables between discharge and death, stable and adverse groups where appropriate. Youden's J statistic (sum of sensitivity and specificity) on receiver operating characteristic (ROC) curve analysis was used to determine the optimum cutoff for age and CT severity score.

Comparison with p values < 0.05 between discharge and death, stable and adverse groups were defined as potential variates and then included in subsequent multivariable analyses. Considering the number of events in our study (death, n = 33, adverse outcomes, n = 55), 7 complete variables were selected for regression analysis based on previous findings and exploration variables to avoid overfitting in the model. Previous studies have shown that critically ill or fatal cases occurred more frequently in older males with comorbidity and higher CT severity scores, while non-critical or surviving patients had fewer observed lymphopenia 9-13,16,17. Besides, most patients also demonstrated abnormal leukocyte 16, but some other laboratory indicators, such as D-dimer, interleukin-6, and lactate dehydrogenase, may not be available in an emergency. Moreover, the incidence of pleural effusion was significantly higher in critically ill COVID-19 patients 18. Multivariable Cox regression model was used to calculate the hazard ratio (HR), 95% confidence interval (CI). A final model selection was performed via a forward stepdown selection process. Power of sample size was calculated by Cox regression power analysis. Kaplan-Meier plots and log-rank test were used to compare the cumulative event rate of adverse clinical outcomes between groups. The area under curve (AUC) of the risk prediction model was obtained by time-correlated ROC analysis. A p value < 0.05 was considered as statistically significant (two-tailed).

## Results

### Patient Characteristics

This study included 703 laboratory-confirmed patients with a mean age of 46.1 years (SD 15.2) (range from 2 months to 86 years old), and 382 (54%) were males, 321 (46%) were females. Comorbidity presented in 201 (29%) patients, of whom 70% (140/201) had only one comorbidity. Twenty patients (3%) were asymptomatic and 593 (84%) patients complained of fever, of whom more than half (51%, 301/593) had high fever (> 38℃). Cough was also a common symptom at onset (71%, 497/703), followed by fatigue (33%, 235/703) and myalgia (15%, 107/703). The median time (IQR) interval between the symptom onset and first chest CT scans, admission, first positive RT-PCR were 5 (2-9), 5 (2-7), and 5 (2-8) days, respectively.

At data cutoff, 659 of the total 703 patients (94%) were discharged from the hospital, 33 patients (5%) died, and the remaining 11 patients (2%) were still hospitalized in ICU. Of the entire cohort, 648 (92%) discharged without any adverse outcome (stable group), 55 (8%) patients developed adverse clinical outcomes (adverse group), of whom 46 (84%) were admitted to the ICU and 20 (36%) required IMV besides death. The median time (IQR) interval between admission and discharge/adverse outcomes was 16 (11-20) days.

Compared with discharge and stable groups, patients in death and adverse groups were older (both p < 0.001) and tended to be males (both p < 0.05). Of note, death and adverse groups had a higher rate to present with comorbidity, especially with more than one comorbidities (all p < 0.001) compared with discharge and stable groups, and the adverse outcomes occurred in a shorter median time (IQR) (14 7-19 vs. 17 12-21, p=0.023, 4 1-12 vs. 17 12-21, p < 0.001) (**Table 1**).

As for laboratory results, patients in death and adverse groups had a higher rate of leukocytosis, neutrophilia, lymphopenia, thrombopenia and showed increase of D-dimer, lactate dehydrogenase, C-reactive protein and decrease of albumin (all p < 0.05) , compared with the discharge and stable groups (**Table 1**).

### CT Imaging Findings

Of the 703 confirmed patients, 52 (7%) patients demonstrated no COVID-19-related pneumonia on initial chest CT. As for the remaining 651 patients, most developed multifocal lesions showing a prominent subpleural distribution as well as bilateral lung and lower lobe involvement predilection (**Table 2**). The most common pattern seen on chest CT was GGO with consolidation (82%, 532/651). More lesions showed mixed morphology (50%, 325/651) instead of pure round morphology (10%, 68/651). Less common CT findings included pleural effusion (3%, 17/703), fibrosis (0.7%, 5/703), pulmonary emphysema (0.3%, 2/703), and pulmonary edema (0.1%, 1/703) (**Table 2**).

Compared with discharge and stable groups, death and adverse groups showed more lung lobes involvement (both p < 0.05). The median CT severity score and proportion of diffuse lesion in death and adverse groups were also higher than discharge and stable groups (both p < 0.001) (**Table 2**). It was worth noting that, although GGO with consolidation was mainly manifestation in all groups, more pure GGO as well as pure round morphology (all p < 0.05) appeared in death and adverse groups, which suggested that we should not ignore patients presenting with a single imaging pattern. In addition, more patients in death and adverse groups presented crazy paving patterns and pleural effusion (all p < 0.05) compared with discharge and stable groups (**Table 2**).

### Relations between Clinical Characteristics, Laboratory Results, CT Imaging Features and Adverse Outcomes on Multivariable Cox Analysis

**Figure 3:**
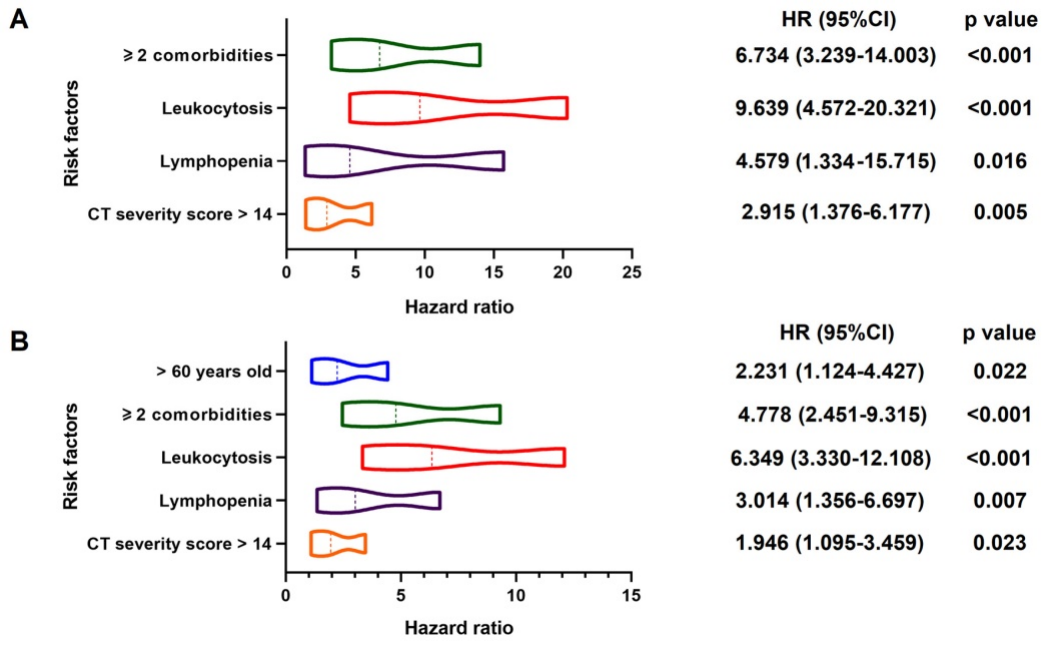
** Predictors of In-hospital Death and Adverse Outcomes.** Positive estimated effect sizes of variates in multivariable Cox regression for death (Panel A: 31/545) and adverse outcomes (Panel B: 49/552). The forest plot displays estimated effect sizes of regression coefficients with 95% CI (x-axis). **Panel A:** Variates associated with a significant increase in in-hospital death were ≥ 2 comorbidities, leukocytosis, lymphopenia and higher CT severity score (> 14). **Panel B:** Variates associated with a significant increase in adverse outcomes were higher age (> 60), ≥ 2 comorbidities, leukocytosis, lymphopenia and higher CT severity score (> 14). HR: hazard ratio; CI: confidence interval.

In multivariable Cox regression model, ≥ 2 comorbidities (HR, 6.734; 95% CI; 3.239-14.003, p < 0.001), leukocytosis (HR, 9.639; 95% CI, 4.572-20.321, p < 0.001), lymphopenia (HR, 4.579; 95% CI, 1.334-15.715, p = 0.016) and CT severity score > 14 (HR, 2.915; 95% CI, 1.376-6.177, p = 0.005) were associated with increased odds of death. And older age (> 60 years) (HR, 2.231; 95% CI, 1.124-4.427, p = 0.022), ≥ 2 comorbidities (HR, 4.778; 95% CI; 2.451-9.315, p < 0.001), leukocytosis (HR, 6.349; 95% CI; 3.330-12.108, p < 0.001), lymphopenia (HR, 3.014; 95% CI; 1.356-6.697, p = 0.007) and CT severity score > 14 (HR, 1.946; 95% CI; 1.095-3.459, p = 0.023) were associated with increased odds of adverse outcomes in multivariable Cox regression model (**Figure 3**). The power of the sample size of ≥ 2 comorbidities, leukocytosis, lymphopenia, CT severity score > 14 in death group were 0.85, 0.78, 0.98, 0.76, respectively and older age (> 60 years), ≥ 2 comorbidities, leukocytosis, lymphopenia, CT severity score > 14 in adverse group were 0.51, 0.87, 0.82, 0.96, 0.54, respectively.

**Figure 4:**
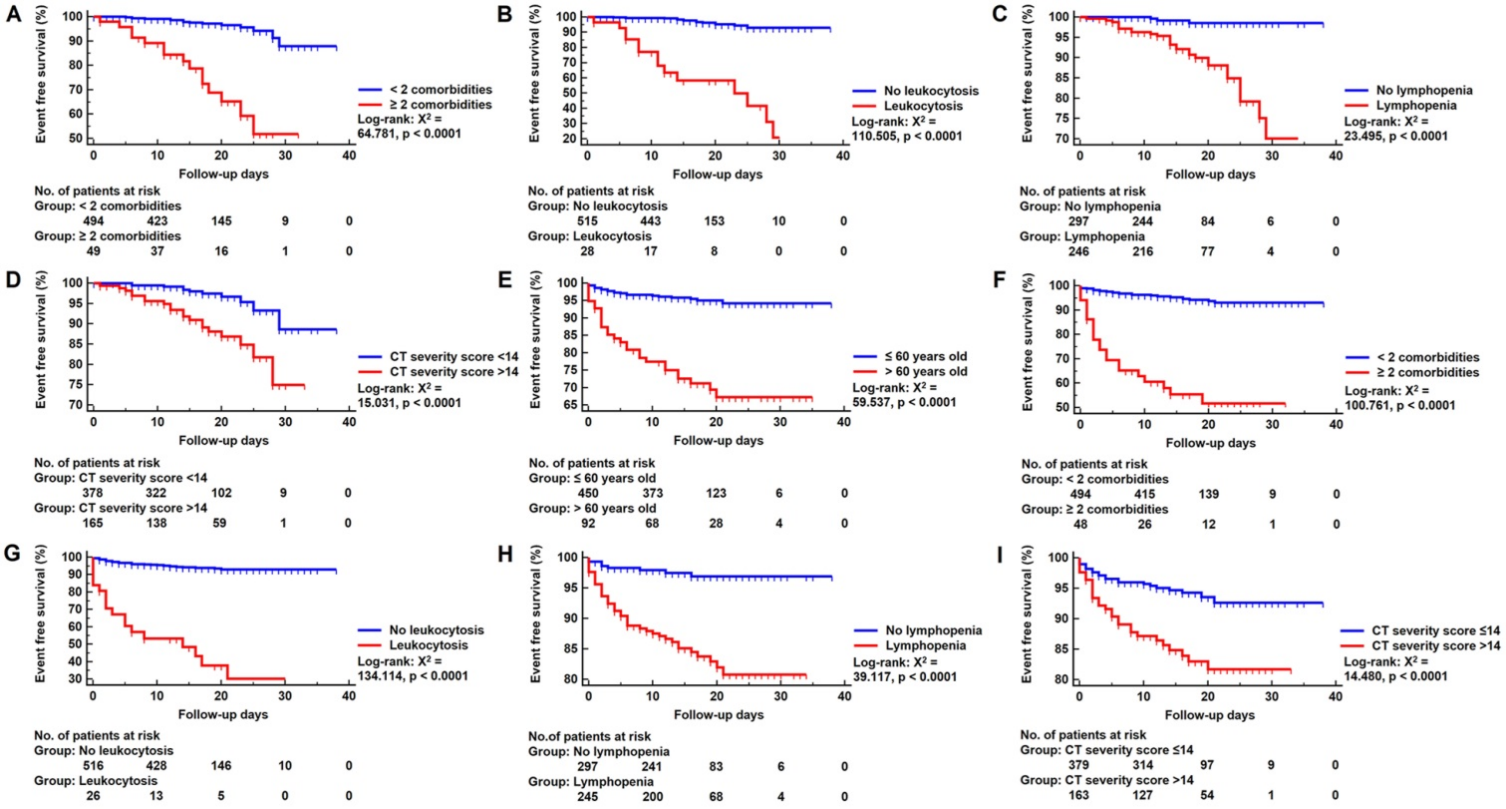
** Kaplan-Meier Survival Curve. Panels A-D:** Kaplan-Meier survival curve for in-hospital death group according to risk factors. **Panels E-I:** Kaplan-Meier survival curve for adverse outcomes group according to risk factors. No.: Number.

As depicted in **Figure 4**, Kaplan-Meier curves with log-rank test were generated for cumulative event rates of death and adverse outcomes groups. ≥ 2 comorbidities (log-rank: X^2^ = 64.781, p < 0.0001), leukocytosis (log-rank: X^2^ = 110.505, p < 0.0001), lymphopenia (log-rank: X^2^ = 23.495, p < 0.0001) and CT severity score >14 (log-rank: X^2^ = 15.031, p < 0.0001) significantly improved the performance of predicting death patients. Similarly, > 60 years old (log-rank: X^2^ = 59.537, p < 0.0001), ≥ 2 comorbidities (log-rank: X^2^ = 100.761, p < 0.0001), leukocytosis (log-rank: X^2^ = 134.114, p < 0.0001), lymphopenia (log-rank: X^2^ = 39.117, p < 0.0001) and CT severity score >14 (log-rank: X^2^ = 14.480, p < 0.0001) significantly improved the performance of predicting adverse outcome patients.

### The Discriminatory Value of Clinical, Laboratory and CT Imaging Features in Risk Prediction Model

**Figure 5:**
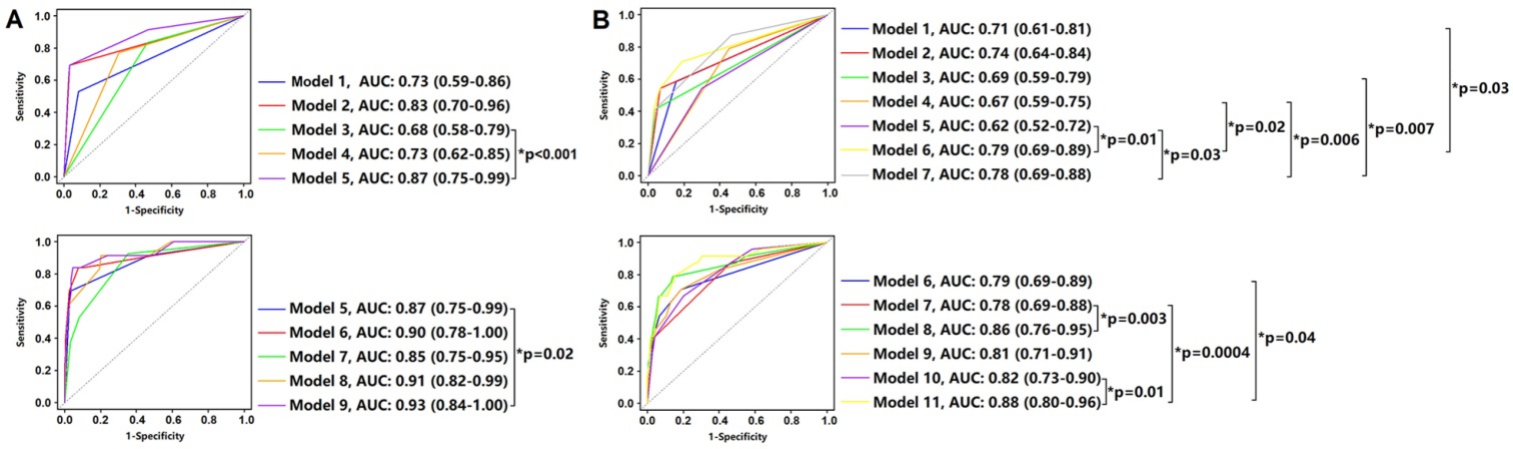
** Prognostic Value of Clinical, Laboratory and CT Imaging Characteristics.** Comparison of time-dependent ROC curves of each as well as combination variable in death (Panel A: at median follow-up 14 days) and adverse outcome (Panel B: at median follow-up 4 days) groups. **Panel A:** Model 1: ≥ 2 comorbidities; Model 2: leukocytosis; Model 3: lymphopenia; Model 4: CT severity score > 14; Model 5: leukocytosis + lymphopenia; Model 6: ≥ 2 comorbidities + leukocytosis + lymphopenia; Model 7: ≥ 2 comorbidities + CT severity score > 14; Model 8: leukocytosis + lymphopenia + CT severity score > 14; Model 9: ≥ 2 comorbidities + leukocytosis + lymphopenia + CT severity score > 14. Compared with Model 3, the combination of leukocytosis and lymphopenia (Model 5) improved the performance in predicting death patients (AUC: 0.68 vs. 0.87, p < 0.001). Similarly, compared with Model 5, the combination of ≥ 2 comorbidities, leukocytosis, lymphopenia and CT severity score > 14 (Model 9) improved the performance in predicting death patients (AUC: 0.87 vs. 0.93, p = 0.02). **Panel B:** Model 1: age > 60 years; Model 2: ≥ 2 comorbidities; Model 3: leukocytosis; Model 4: lymphopenia; Model 5: CT severity score > 14; Model 6: age > 60 years + ≥ 2 comorbidities; Model 7: leukocytosis + lymphopenia; Model 8: age > 60 years + ≥ 2 comorbidities + leukocytosis + lymphopenia; Model 9: age > 60 years + ≥ 2 comorbidities + CT severity score > 14; Model 10: leukocytosis + lymphopenia + CT severity score > 14; Model 11: age > 60 years + ≥ 2 comorbidities + leukocytosis + lymphopenia + CT severity score > 14. Compared with Model 10, the combination of age > 60 years, ≥ 2 comorbidities, leukocytosis, lymphopenia, CT severity score > 14 (Model 11) improved the performance in predicting adverse outcome patients (AUC: 0.82 vs. 0.88, p = 0.01). ROC: receiver operating characteristic; AUC: area under curve.

The prognostic values of clinical, laboratory and CT features for death and adverse outcomes are shown in **Figure 5**. After median follow-up of 14 days, the combination of ≥ 2 comorbidities, leukocytosis, lymphopenia and CT severity score > 14 (Model 9) showed the highest performance in predicting death patients with AUC of 0.87 (**Figure 5A**), while the combination of age > 60 years, ≥ 2 comorbidities, leukocytosis, lymphopenia, CT severity score > 14 (Model 11) showed the highest performance in predicting adverse outcome patients with AUC of 0.88 after median follow-up of 4 days (**Figure 5B**).

## Discussion

As the number of patients with SARS-CoV-2 infections is rapidly growing worldwide, but specialized treatment is not yet available, early identification of patients at risk of becoming critically ill is essential for timely intervention and active intensive care. Previous studies revealing some risk factors associated with death were mainly skewed in Wuhan, the epicenter of China 9-12. To the best of our knowledge, this multicenter study had the largest sample size from 8 provinces in China with clear available prognosis outcomes. We enrolled 703 patients hospitalized with laboratory confirmed COVID-19 and identified that multiple comorbidities, leukocytosis, lymphopenia and higher CT severity scores on admission were associated with higher rates of in-hospital death, while adverse outcomes were associated with older age, multiple comorbidities, leukocytosis, lymphopenia and higher CT severity scores.

Although it is difficult to calculate the final true mortality of COVID-19 as the epidemic is still continuing, the large case series involving 72,314 patients provided by the Chinese Centers for Disease Control and Prevention showed that there were no deaths among cases ≤ 9 years old but the case-fatality rate (CFR) of patients between 70 and 79 years old and patients ≥ 80 years old was as high as 8% and 14.8%, respectively 3. The same phenomenon was also happening in Italy, where patients over 70 years old, especially those older than 80 years, had a higher mortality rate of 20.2% 19. Our data further supported that > 60 years old was a predictor for adverse outcomes. In addition, females may be less susceptible to the virus due to the protective effects of the X chromosome and sex hormones 20, consistent with current study finding more COVID-19 infections in males than females 16. The expression level of angiotensin-converting enzyme 2 (ACE2), the functional receptor for SARS-CoV-2, was found higher in males than that in females, which may be another explanation 21. Different to these findings, the number of male infectors was basically equal to that of females in our study. And, interestingly, female was found to be protective against death and adverse outcomes, suggesting that susceptibility factors in males may also be the reasons for severity.

At the same time, several reports concerning co-morbid disease with COVID-19 prompted that adequate attention should be paid to comorbidity 12,16,22. Data showed that the CFR of COVID-19 patients with cardiovascular disease, diabetes, chronic respiratory disease, hypertension, and cancer was 10.5%, 7.3%, 6.3%, 6.0%, and 5.6%, respectively 3. In our study, the most common comorbidity were hypertension (17%, 118/703), followed by diabetes (9%, 64/703) and cardiovascular disease (5%, 35/703). We further revealed that the presence of ≥ 2 comorbidities was associated with an approximately 5-fold time increase in the risk of death as well as adverse outcomes. Further and detailed evaluation of the impact of different comorbidities on patients with COVID-19 is necessary and of great value to guiding proper inter-disciplinary management, especially for the elderly patients.

For example, recent studies have put an emphasis on cardiovascular disease (CVD) complications showing that patients with severe cardiovascular damage and underlying cardiac insufficiency was associated with adverse events 11,12,21. There may be interactions and synergies between acute pulmonary infection and cardiovascular disease. Systemic inflammatory response may trigger the rupture and erosion of coronary plaques, and patients with heart injury are more likely to develop myocardial ischemia or cardiac dysfunction after severe pneumonia 23. Both COVID-19 and heart failure can cause hypoxemia and eventually lead to death. Similarly, worsening heart disease may exacerbate the management of COVID-19 disease. Of note, ACE2, the receptor for SARS-CoV-2 infection, is also highly expressed in heart as a gatekeeper to the renin-angiotensin-aldosterone system (RAAS) and has protective effects on CVDs 21,24. Upregulating the expression or preventing ACE2 loss is one of the confirmed mechanisms of angiotensin-converting enzyme inhibitor (ACEI) and angiotensin receptor blocker (ARB) therapy 25, which is now widely used in patients with CVDs. However, ACEI/ARB therapy was considered to have the probability of increasing the risk of SARS-CoV-2 infection 24, but it is needed to clarify whether ACE2 could upregulate viral load and further result in disease severity to identify the multifaceted role of the ACE2 in COVID-19. Thus, a lot of puzzles remain to be uncovered.

As for laboratory findings, the hallmark laboratory findings of COVID-19 included lymphopenia. Yang et al. found that more than 80% (44/52) of critically ill patients developed lymphopenia 26. Similar to SARS-CoV, SARS-CoV-2 may act primarily on lymphocytes, especially T lymphocytes, and destroy their cytoplasmic component, leading to suppression of cellular immune function 16,27. Lymphocyte tracing showed that the lymphocyte counts of survivors were the lowest on the 7th day after symptom onset, while non-survivors suffered from severe lymphopenia until death 9. Thus, the level of lymphopenia may reflect the severity of infection in patients with COVID-19. Current research generally suggested that patients with COVID-19 have normal or low leukocyte counts.^9^ However, interestingly, although 20% (131/641) of the total population showed leukocytopenia in our study, the death and adverse groups showed more patients with leukocytosis compared with the discharge and stable groups, which was also consistent with previous studies 12,16. Some patients, especially those severe cases, demonstrated a mixed infection of bacteria and fungi 16, which may be the cause of leukocytosis. Study found that mixed bacterial infection in the context of viral pneumonia was a predictor of poor outcomes 28, thought to be related to dysregulation of T cells, antigen-specific T cells and plasma cytokine levels 29. Besides, the proposition of cytokine release syndrome (CRS) has also caused widespread concern from the scientific community. Novel coronavirus may activate CD4+ T lymphocytes, produce granulocyte-macrophage colony-stimulating factor (GM-CSF), and induce monocytes such as CD14^+^, CD16^+^, with high expression of IL-6, to accelerate the inflammatory response, thereby forming a cytokine storm and resulting in severe immune injury 30,31. Accordingly, it is of great significance to elaborate on lymphopenia and leukocytosis and further explore the changes of various inflammatory factors.

CT severity score was implemented in many previous studies to semi-quantitatively evaluate the lung involvement of COVID-19 which is assigned on basis of the extent all abnormal lung areas involved, sharing basic principle with the CT severity score system we proposed. Feng et al. found that the CT severity score was related to the inflammatory levels and higher CT severity on admission was a risk factor for short-term progression of patients with COVID-19 outside Wuhan 17. Similarly, our study also found that CT severity score > 14 on admission was one of predictors of in-hospital death as well as adverse outcomes. In addition, some researchers revealed predictive capability of CT features 32,33, while others demonstrated the relationship between CT findings as well as disease course, proving that serial CT imaging was helpful for monitoring the dynamic changes of disease and sensitively reflecting the treatment effect 34,35. However, we cannot ignore the fact that the pneumonia of COVID-19 lacks specificity in imaging findings despite its value of predicting death and monitoring disease progression and larger-scale cohorts are necessary for verification. No association between pleural effusion and death or adverse outcomes was found at our multivariable Cox analysis, although more patients developed pleural effusion in critically ill COVID-19 patients 18.

It was worth noting that, in addition to risk factors mentioned in this study, higher lactate dehydrogenase, procalcitonin > 0.5 ng / ml, aspartate aminotransferase > 40U / liter, higher D-dimer were also considered to be associated with an increased risk of in-hospital mortality in other studies 9,10,12,13. The diverse observations from different clinical studies may be attributed to insufficient adjustments for important confounding factors such as age, sex as well as health status of patients, differences in sample size, and inconsistent statistical methods. Besides, retrospective studies inevitably have incomplete or inaccurate health records, which exacerbate this bias. Therefore, the large sample study with accurate statistical methods is worth advocating.

Our research had some limitations. First, this retrospective study relied on clinical records. Accordingly, some certain information was missing, and some information relied on patients' memory such as timing of symptom onset and exposure history, which may be affected by recall bias. At the same time, the existence of heterogeneity in some clinical information was inevitable, such as the admission of ICU and the application of IMV. Second, the differences in treatment existed among centers, and we did not consider the effect of treatment on prognosis. Third, although our study had the largest sample size with clear prognosis information, the number of hard endpoints was small, thus, another multicenter study with larger and more representative samples are needed. Last, four well-experienced thoracic radiologists analyzed the CT images in consensus in our study and we did not provide the inter-reader agreement in CT imaging analysis.

## Conclusions

To our knowledge, this largest multicenter retrospective prognostic study to date found that older age, multiple comorbidities, leukocytosis, lymphopenia and higher CT severity score were risk factors for in-hospital death or adverse outcomes in laboratory confirmed COVID-19 patients. These findings may bring light and hope for combating against the COVID-19 in the epidemic.

## Supplementary Material

Supplementary figures and tables.

## Figures and Tables

**Table 1:** Clinical Characteristics and Laboratory Findings of Patients with COVID-19

Characteristic	All patients(n = 703)	Discharge group (n = 659)	Death group (n = 33)	* p value	Stable group (n = 648)	Adverse group (n = 55)	# p value
**Clinical characteristics** **Age, years (SD)**	46.1 ± 15.2	45.0 ± 14.6	64.7 ± 13.4	< 0.001	44.9 ± 14.3	60.5 ± 17.2	< 0.001
**Sex- no. (%)**				0.026			0.022
Male	382 (54, 382/703)	349 (53, 349/659)	24 (73, 24/33)		344 (53, 344/648)	38 (69, 38/55)	
Female	321 (46, 321/703)	310 (47, 310/659)	9 (27, 9/33)		304 (47, 304/648)	17 (31, 17/55)	
**Exposure history**- no. (%)				0.019			0.003
Exposure to Wuhan	294 (42, 294/703)	284 (43, 284/659)	7 (21, 7/33)		280 (43, 280/648)	14 (25, 14/55)	
Exposure to confirmed patients	199 (28, 199/703)	188 (29, 188/659)	10 (30, 10/33)		185 (29, 185/648)	14 (25, 14/55)	
Unknown exposure	210 (30, 210/703)	187 (28, 187/659)	16 (48, 16/33)		183 (28, 183/648)	27 (49, 27/55)	
**Occupation**- no. (%)				0.741			1.00
Hospital staff	15 (2, 15/703)	14 (2, 14/659)	1 (3, 1/33)		14 (2, 14/648)	1 (2, 1/55)	
Non-hospital staff	688 (98, 688/703)	645 (98, 645/659)	32 (97, 32/33)		634 (98, 634/648)	54 (98, 54/55)	
**Symptoms**- no. (%)							
Fever	593 (84, 593/703)	532 (84, 556/659)	26 (79, 26/33)	0.392	547 (84, 547/648)	45 (82, 45/55)	0.612
<37.3℃	157 (22, 157/703)	147 (22, 147/659)	9 (27, 9/33)		145 (22, 145/648)	12 (22, 12/55)	
37.3-38.0℃	231 (33, 231/703)	220 (33, 220/659)	9 (27, 9/33)		219 (34, 219/648)	12 (22, 12/55)	
38.1-39.0℃	266 (38, 266/703)	251 (38, 251/659)	12 (36, 12/33)		246 (38, 246/648)	20 (36, 20/55)	
>39℃	35 (5, 35/703)	30 (5, 30/659)	1 (3, 1/33)		28 (4, 28/648)	7 (13, 7/55)	
Cough	497 (71, 497/703)	464 (70, 464/659)	25 (76, 25/33)	0.510	458 (71, 458/648)	38 (69, 38/55)	0.804
Myalgia	107 (15, 107/703)	99 (15, 99/659)	7 (21, 7/33)	0.335	97 (15, 97/648)	10 (18, 10/55)	0.524
Fatigue	235 (33, 235/703)	216 (33, 216/659)	14 (42, 14/33)	0.251	211 (33, 211/648)	23 (42, 23/55)	0.162
Headache	60 (9, 60/703)	56 (8, 56/659)	3 (9, 3/33)	1.00	55 (9, 55/648)	5 (9, 5/55)	1.00
Nausea	47 (7, 47/703)	43(7, 43/659)	3 (9, 3/33)	0.826	40 (6, 40/648)	7 (13, 7/55)	0.112
Abdominal pain	10 (1, 10/703)	10 (2, 10/659)	0 (0, 0/33)	0.321	8 (14, 8/56)	2 (4, 2/55)	0.218
Diarrhea	58 (8, 58/703)	53 (8, 53/659)	2 (6, 2/33)	0.935	50 (8, 50/648)	8 (15, 8/55)	0.130
Dyspnea	86 (12, 86/703)	58 (9, 58/659)	18 (55, 18/33)	< 0.001	54 (8, 54/648)	31 (56, 31/55)	< 0.001
No obvious symptoms	20 (3, 20/703)	18 (3, 18/659)	1 (6, 2/33)	0.326	18 (3, 18/648)	2 (4, 2/55)	1.00
*^13, 14^ Clinical severity- no. (%)				< 0.001			< 0.001
Mild	21 (3, 21/703)	21 (3, 21/659)	0 (0, 0/33)		21 (3, 21/648)	0 (0, 0/55)	
Moderate	575 (82, 575/703)	575 (87, 575/659)	0 (0, 0/33)		573 (88, 573/648)	2 (4, 2/55)	
Severe	62 (9, 62/703)	52 (8, 52/659)	5 (15, 5/33)		50 (8, 50/648)	12 (22, 12/55)	
Critical	45 (6, 45/703)	11 (2, 11/659)	28 (85, 28/33)		4 (1, 4/648)	41 (75, 41/55)	
**Comorbidity**- no. (%)	201 (29, 201/703)	167 (25, 167/659)	25 (76, 25/33)	< 0.001	160 (25, 160/648)	41 (75, 41/55)	< 0.001
Cardiovascular disease	35 (5, 35/703)	20 (3, 20/659)	12 (36, 12/33)	< 0.001	20 (3, 20/648)	15 (27, 15/55)	< 0.001
Diabetes	64 (9, 64/703)	49 (7, 49/659)	12 (36, 12/33)	< 0.001	46 (7, 46/648)	18 (33, 18/55)	< 0.001
Hypertension	118 (17, 118/703)	93 (14, 93/659)	17 (52, 17/33)	< 0.001	89 (14, 89/648)	30 (53, 29/55)	< 0.001
COPD	13 (2, 13/703)	9 (1, 9//659)	4 (12, 4/33)	0.002	9 (1, 9/648)	4 (7, 4/55)	0.010
Chronic liver disease	29 (4, 29/703)	27 (4, 27/659)	1 (3, 1/33)	1.0	26 (4, 26/648)	3 (5, 3/55)	0.870
Chronic kidney disease	10 (1, 10/703)	6 (1, 6/659)	3 (9, 3/33)	0.006	3 (1, 3/648)	7 (13, 7/55)	< 0.001
Malignancy	9 (1, 9/703)	8 (1, 8/659)	1 (3, 1/33)	0.439	6 (1, 6/648)	3 (5, 3/55)	0.027
**Number of comorbidities**- no. (%)				< 0.001			< 0.001
0	502 (71, 502/703)	492 (75, 492/659)	8 (24, 8/33)		488 (75, 488/648)	14 (25, 14/55)	
1	140 (20, 140/703)	125 (19, 125/659)	10 (30, 10/33)		123 (19, 123/648)	17 (31, 17/55)	
2	50 (7, 50/703)	39 (6, 39/659)	9 (27, 9/33)		35 (5, 35/648)	15 (27, 15/55)	
3	8 (1, 8/703)	3 (0.5, 3/659)	4 (12, 4/33)		2 (0.3, 2/648)	6 (11, 6/55)	
4	1 (0.1, 1/703)	0 (0, 0/659)	0 (0, 0/33)		0 (0, 0/648)	1 (2, 1/55)	
5	2 (0.3, 2/703)	0 (0, 0/659)	2 (6, 2/33)		0 (0, 0/648)	2 (4, 2/55)	
**The interval between symptoms onset and the first CT scan (d) (IQR)**	5 (2-9)	5 (2-9)	6 (3-9)	0.762	5 (2-9)	6 (3-9)	0.775
**The interval between symptoms onset and admission (d) (IQR)**	5 (2-7)	5 (2-7)	6 (4-7)	0.319	5 (2-7)	6 (3-7)	0.244
**The interval between symptoms onset and the first positive RT-PCR (d) (IQR)**	5 (2-8)	4 (1-8)	7 (0-10)	0.170	4 (1-8)	7 (4-10)	0.002
**The interval between admission and discharge / adverse outcome (d) (IQR)**	16 (11-20)	17 (12-21)	14 (7-19)	0.023	17 (12-21)	4 (1-12)	< 0.001
**Clinical outcomes** - no. (%)							
Adverse outcomes	55 (8, 55/703)	11 (2, 11/659)	33 (100, 33/33)	< 0.001	0 (0, 0/648)	55 (100, 55/55)	NA
Death	33 (5, 33/703)	0 (0, 0/659)	33 (100, 33/33)	NA	0 (0, 0/648)	32 (58, 32/55)	NA
ICU admission	46 (7, 46/703)	11 (2, 11/659)	24 (73, 24/33)	< 0.001	0 (0, 0/648)	46 (84, 46/55)	NA
IMV	20 (3, 20/703)	4 (0.6, 4/659)	14 (42, 14/33)	< 0.001	0 (0, 0/648)	20 (36, 20/55)	NA
**Laboratory results**							
**Blood routine**- no. (%)							
Leucocytes				< 0.001			< 0.001
Increased	39 (6, 39/641)	18 (3,18/597)	16 (47, 16/33)		17 (3, 17/586)	22 (40, 22/55)	
Decreased	131 (20, 131/641)	128 (21, 128/597)	2 (6, 2/33)		125 (21, 125/586)	6 (11, 6/55)	
Neutrophils				< 0.001			< 0.001
Increased	60 (11, 60/552)	42 (8, 42/514)	15 (50, 15/30)		40 (8, 40/503)	20 (43, 21/49)	
Decreased	90 (16, 90/552)	87 (17, 87/514)	2 (7, 2/30)		85 (17, 85/503)	5 (10, 5/49)	
Lymphopenia	271 (47, 271/571)	238 (45, 238/533)	28 (90,28/31)	< 0.001	230 (44, 230/522)	41 (84, 41/49)	< 0.001
NLR	2.3 (1.5-4.0)	2.2 (1.4-3.7)	6.9 (2.6-22.0)	< 0.001	2.2 (1.4-3.6)	5.1 (2.5-14.3)	< 0.001
Platelets				0.001			< 0.001
Increased	25 (5, 25/555)	25 (5, 25/518)	0 (0, 0/29)		24 (5, 24/509)	1 (2, 1/46)	
Decreased	119 (21, 119/555)	101 (19, 101/518)	14 (48, 14/29)		98 (19, 98/509)	21 (46, 21/46)	
Hemoglobin (Decreased)	195 (35, 195/565)	182 (35, 182/526)	10 (34, 10/29)	0.743	177 (34, 177/516)	18 (37, 18/49)	0.942
**Coagulation function**- no. (%)							
Activated partial thromboplastin time				0.303			0.081
Increased	110 (30, 110/369)	108 (31, 108/350)	1 (20, 3/15)		107 (31, 107/343)	3 (12, 3/26)	
Decreased	6 (2, 6/369)	5 (1, 5/350)	1 (7, 1/15)		5 (1, 5/343)	1 (4, 1/26)	
Prothrombin time				0.031			0.025
Increased	138 (32, 138/434)	122 (30, 122/405)	12 (55, 12/22)		119 (30, 119/396)	19 (50, 19/38)	
Decreased	3 (1, 3/434)	3 (0.7, 3/405)	0 (0, 0/22)		3 (1, 3/396)	0 (0, 0/38)	
D-dimer (Increased)	114 (30, 114/378)	95 (27, 95/350)	15 (71, 15/21)	< 0.001	90 (26, 90/342)	24 (67, 24/36)	< 0.001
**Blood biochemistry**- no. (%)							
Albumin (Decreased)	260 (45, 260/581)	231 (43, 231/542)	21 (70, 21/30)	< 0.001	225 (42, 225/533)	35 (73, 35/48)	< 0.001
Alanine aminotransferase (Increased)	95 (19, 95/504)	79 (17, 79/474)	10 (43, 10/23)	0.013	76 (16, 76/467)	19 (51, 19/37)	< 0.001
Aspartate aminotransferase (Increased)	82 (18, 82/460)	66 (15, 66/433)	13 (57, 13/23)	< 0.001	64 (15, 64/426)	18 (53, 18/34)	< 0.001
Total bilirubin (Increased)	60 (14, 60/430)	49 (12, 49/409)	11 (55, 11/20)	< 0.001	47 (12, 47/402)	13 (46, 13/28)	< 0.001
Serum creatinine				< 0.001			< 0.001
Increased	80 (16, 80/485)	57 (13, 57/454)	19 (70, 19/27)		53 (12, 53/445)	26 (65, 26/40)	
Decreased	129 (27, 129/485)	126 (28, 126/454)	3 (11, 3/27)		123 (28, 123/445)	6 (15, 6/40)	
Creatine kinase				0.004			< 0.001
Increased	47 (12, 47/394)	37 (10, 37/370)	8 (38, 8/21)		35 (10, 35/365)	12 (41, 12/29)	
Decreased	93 (24, 93/394)	90 (24, 90/370)	3 (14, 3/21)		89 (24, 89/365)	4 (14, 4/29)	
Lactate dehydrogenase (Increased)	171 (45, 171/383)	144 (41, 144/354)	22 (92, 22/24)	< 0.001	138 (40, 138/347)	33 (92, 33/36)	< 0.001
Myoglobin (Increased)	33 (18, 33/181)	19 (12, 19/160)	13 (65, 13/20)	< 0.001	16 (10, 16/157)	17 (71, 17/24)	< 0.001
**Infection-related biomarkers**- no. (%)							
Procalcitonin (Increased)	259 (56, 259/462)	238 (55, 238/434)	17 (81, 17/21)	0.019	234 (55, 234/426)	25 (69, 25/36)	0.092
Interleukin-6 (Increased)	25 (37, 25/68)	25 (37, 25/67)	0 (0, 0 /1)	0.336	25 (37, 25/67)	0 (0, 0/1)	0.336
Erythrocyte sedimentation rate (Increased)	227 (68, 227/333)	211 (68, 211/311)	14 (74, 14/19)	0.596	208 (68, 208/306)	19 (70, 19/27)	0.798
C-reactive protein (Increased)	409 (70, 409/587)	376 (68, 376/553)	27 (96, 27/28)	0.001	370 (68, 370/544)	39 (91, 39/43)	0.002

Data are given as mean (SD), n (%) or median (IQR). The normal range refers to the criteria of each hospital. Increased means over the upper limit of the normal range and decreased means below the lower limit of the normal range. * p value is statistics of comparison between discharge and death groups while # p value is statistics of comparison between stable and adverse groups. *^13,14^ Clinical severity: according to the clinical manifestations based on the Diagnosis and Treatment Program of 2019 New Coronavirus Pneumonia (trial sixth version), confirmed patients were divided into (1) mild: mild clinical symptoms, without imaging findings of pneumonia; (2) moderate: having symptoms of fever and respiratory symptoms, with imaging findings of pneumonia; (3) severe: meet any of the followings: a. respiratory distress, RR ≥30 times/min; b. SpO_2_ <93% at rest; c. PaO_2_/FiO_2_ ≤ 300 mmHg and critical types; (4) critical: meet any of the followings: a. respiratory failure, need mechanical assistance; b. shock; c. “extra pulmonary” organ failure, intensive care unit is needed. SD: standard deviation; IQR: median and interquartile range; COVID-19: Coronavirus Disease-19; COPD: chronic obstructive pulmonary disease; RT-PCR: reverse-transcription-polymerase-chain-reaction; ICU: intensive care unit; IMV: invasive mechanical ventilation support; NA: not available; NLR: neutrophil-to-lymphocyte ratio; RR: respiratory rate; SpO_2_: oxygen saturation; PaO_2_: partial pressure of oxygen; FiO_2_: fraction of inspired oxygen.

**Table 2:** CT Imaging Findings in 703 Patients with COVID-19

Findings	All patients (n = 703)	Discharge group (n =559)	Death group (n = 33)	*p value	Stable group (n = 648)	Adverse group (n = 55)	#p value
**Negative-** no. (%) **Positive-** no. (%)	52 (7, 52/703)	49 (9, 49/559)	2 (6, 2/33)	1.00	49 (8, 49/648)	3 (5, 3/55)	0.760
	651 (93, 651/703)	610 (92, 610/559)	31 (94, 31/33)		599 (92, 599/648)	52 (95, 52/55)	
Unilateral lung- no. (%) Bilateral lung- no. (%)	101 (16, 101/651)	99 (16, 99/610)	1 (3, 1/31)	0.091	97 (16, 97/599)	4 (8, 4/52)	0.104
	550 (84, 550/651)	511 (84, 511/610)	30 (97, 30/31)		502 (84, 502/599)	48 (92, 48/52)	
Single lesion- no. (%) Multiple lesions- no. (%)	40 (6, 40/651)	39 (6, 39/610)	1 (3, 1/31)	0.741	39 (7, 39/599)	1 (2, 1/52)	0.308
	611 (94, 611/651)	571 (94, 571/610)	30 (97, 30/31)		560 (94, 560/599)	51 (98, 51/52)	
**Number of involved lobes** - no. (%)				0.001			0.005
One	72 (11, 72/651)	71 (12, 71/610)	1 (3, 1/31)		70 (12, 70/599)	2 (4, 2/52)	
Two	82 (13, 82/651)	80 (13, 80/610)	0 (0, 0/31)		79 (13, 79/599)	3 (6, 3/52)	
Three	94 (14, 94/651)	91 (15, 91/610)	2 (6, 2/31)		91 (15, 91/599)	3 (6, 3/52)	
Four	119 (18, 119/651)	104 (17, 104/610)	13 (42, 13/31)		102 (17, 102/599)	17 (33, 17/52)	
Five	284 (44, 284/651)	264 (43, 264/610)	15 (48, 15/31)		257 (43, 257/599)	27 (52, 27/52)	
**Involved lung zones**- no. (%)							
Upper lobe	545 (84, 545/651)	507 (83, 507/610)	30 (97, 30/31)	0.027	496 (83, 496/599)	49 (94, 49/52)	0.032
Middle lobe	427 (66, 427/651)	391 (64, 391/610)	26 (84, 26/31)	0.011	382 (64, 382/599)	45 (87, 45/52)	0.001
Lower lobe	614 (94, 614/651)	576 (94, 576/610)	29 (94, 29/31)	0.881	564 (94, 564/599)	50 (96, 50/52)	0.776
**Predominant distribution** - no. (%)							
Upper lobe of right lung	394 (60, 394/651)	370 (61, 370/610)	19 (61, 19/31)	0.761	361 (60, 361/599)	33 (63, 33/52)	0.651
Middle lobe of right lung	427 (66, 427/651)	391 (64, 391/610)	26 (84, 26/31)	0.011	382 (64, 382/599)	45 (87, 45/52)	0.001
Lower lobe of right lung	564 (87, 564/651)	526 (86, 526/610)	29 (94, 29/31)	0.166	515 (86, 515/599)	49 (94, 49/52)	0.093
Upper lobe of left lung	491 (75, 491/651)	456 (75, 456/610)	27 (87, 27/31)	0.057	445 (74, 445/599)	46 (88, 46/52)	0.023
Lower lobe of left lung	538 (83, 538/651)	501 (82, 501/610)	29 (94, 29/31)	0.038	491 (82, 491/599)	47 (90, 47/52)	0.124
**Distribution**- no. (%)				< 0.001			< 0.001
Subpleural	511 (78, 511/651)	492 (81, 492/610)	14 (45, 14/31)		483 (81, 483/599)	28 (54, 28/52)	
Diffuse	119 (18, 119/651)	97 (16, 97/610)	17 (55, 17/31)		96 (16, 96/599)	23 (44, 23/52)	
Others	21 (3, 21/651)	21 (3, 21/610)	0 (0, 0/31)		20 (3, 20/599)	1 (2, 1/52)	
**CT severity score (IQR)**	9.0 (4.0-16.0)	9.0 (5.0-16.0)	15.0 (14.0-20.0)	< 0.001	9.0 (5.0-16.0)	15.0 (11.3-20.0)	< 0.001
1-5	190 (29, 190/651)	185 (30, 185/610)	3 (10, 3/31)		183 (31, 183/599)	7 (13, 7/52)	
6-10	158 (24, 158/651)	155 (25, 155/610)	2 (6, 2/31)		154 (26, 154/599)	4 (8, 4/52)	
11-15	126 (19, 126/651)	110 (18, 110/610)	12 (39, 12/31)		107 (18, 107/599)	19 (37, 19/52)	
16-20	177 (27, 177/651)	160 (26, 160/610)	14 (45, 14/31)		155 (26, 155/599)	22 (42, 22/52)	
**Signs - no. (%)**							
Pure GGO	97 (15, 97/651)	83 (14, 83/610)	12 (39, 12/31)	< 0.001	82 (14, 82/599)	15 (29, 15/52)	0.003
Pure consolidation	14 (2, 14/651)	10 (2, 10/610)	3 (10, 3/31)	0.021	10 (2, 10/599)	4 (8, 4/52)	0.018
GGO with consolidation	532 (82, 532/651)	509(83, 509/610)	16 (52, 16/31)	< 0.001	499 (83, 499/599)	33 (63, 33/52)	< 0.001
ILD	514 (79, 514/651)	480 (79, 480/610)	26 (84, 26/31)	0.490	471 (79, 471/599)	43 (83, 43/52)	0.491
Crazy-paving pattern	415 (64, 415/651)	379 (62, 379/610)	27 (87, 27/31)	0.005	373 (62, 373/599)	42 (81, 42/52)	0.008
Pleural effusion	17 (3, 17/651)	7 (1, 7/610)	9 (29, 9/31)	< 0.001	6 (1, 6/599)	12 (21, 11/52)	< 0.001
**Morphology - no. (%)**				0.001			0.006
Pure round morphology	68 (10, 68/651)	57 (9, 57/610)	9 (29, 9/31)		56 (9, 56/599)	12 (23, 12/52)	
Pure irregular morphology	258 (40, 69/651)	242 (40, 242/610)	12 (39, 12/31)		238 (40, 238/599)	20 (38, 20/52)	
Mixed morphology	325 (50, 325/651)	311 (51, 311/610)	10 (32, 10/31)		305 (51, 305/599)	20 (38, 20/52)	

Data are given as n (%) or median (IQR). * p value is statistics of comparison between discharge and death groups while # p value is statistics of comparison between stable and adverse groups. IQR: interquartile range; COVID-19: Coronavirus Disease-19; GGO: ground-glass opacity; ILD: interstitial lung disease.

## Abbreviations

COVID-19: Coronavirus Disease-19
SARS-CoV-2: severe acute respiratory syndrome coronavirus-2
RT-PCR: reverse-transcription-polymerase-chain-reaction
ICU: intensive care unit
IMV: invasive mechanical ventilation support
COPD: chronic obstructive pulmonary disease
GGO: ground-glass opacity
ILD: interstitial lung disease
ROC: receiver operating characteristic
HR: hazard ratio
CI: confidence interval
AUC: area under curve
MERS: Middle East Respiratory Syndrome
SARS: Severe Acute Respiratory Syndrome
CFR: case-fatality rate
ACE2: angiotensin-converting enzyme 2
CVD: cardiovascular disease
RAAS: renin-angiotensin-aldosterone system
ACEI: angiotensin-converting enzyme inhibitors
ARB: angiotensin receptor blockers
SARS-CoV: severe acute respiratory syndrome coronavirus
CRS: cytokine release syndrome
GM-CSF: granulocyte-macrophage colony-stimulating factor
NLR: neutrophil-to-lymphocyte ratio
