# Supplementary material for: Risk factors for adverse clinical outcomes with COVID-19 in China: a multicenter, retrospective, observational study
Source: Theranostics. 2020 May 15;10(14):6372–83. doi: 10.7150/thno.46833 (PMC7255028; doi:10.7150/thno.46833)
Supplement: Supplementary file 1 — Supplementary figures and tables. [file thnov10p6372s1.pdf]

Supplementary Materials

CT Image Acquisition

Chest CT scans were performed using ≥ 16 slice multidetector CT scanners (Aquilion ONE / Aquilion PRIME / BrightSpeed / BrightSpeed S / Brilliance 16 / Brilliance 64 / Discovery CT750 HD / eCT / Fluorospot Compact FD / HiSpeed Dual / iCT 256 / Ingenuity CT / Ingenuity Flex / LightSpeed VCT / LightSpeed 16 / NeuViz 16 Classic / Optima CT520 Series / Optima CT540 / Optima CT680 Series / ScintCare CT 16E / Sensation 64 / SOMATOM Definition AS+ / SOMATOM Definition Flash / uCT 510) without use of iodinated contrast agents. To minimize motion artifacts, patients were asked to hold their breath, then axial CT images were acquired during end-inspiration. The CT scan protocols were as follows: tube voltage, 100-120 kVp; effective tube current, 110-250 mAs; detector collimation, 16-320 x 0.625-2.5 mm; slice thickness, 0.625-2.5 mm; pitch, 0.8-1.375. Based on the raw data, the CT images were reconstructed by iterative reconstruction technique if possible.

CT Image Interpretation

All Digital Imaging and Communications in Medicine (DICOM) images were reviewed by four radiologists (Z.Y.S, L.Q, F. X. and J. Z.) with 18, 6, 5 and 5 years of experiences in thoracic radiology in core lab in Jinling Hospital, Medical School of Nanjing University. They independently assessed the axial CT images and/or multi-planar reconstructed images without access to patient's clinical or laboratory results. The following features of chest thin-slice CT image were recorded: (1) lesion distribution: subpleural, diffuse or others; (2) lesion morphology: round, irregular or mixed; (3) lesion number: single or multiple; (4) main signs: the presence of pure ground-glass opacity (GGO), pure consolidation, GGO with consolidation, interstitial lung disease (ILD), crazy-paving pattern, (5) other abnormalities: fibrosis, pleural effusion, pulmonary emphysema, pulmonary edema; (6) the number of affected lung lobes and lung segments. GGO is defined as a fuzzy increase in lung attenuation without obscuring the underlying blood vessels [1]. Consolidation is defined as increased attenuation of lung parenchyma, blurring the edges of blood vessels and airway walls [2]. ILD is defined as some sparing of individual lobules, forming a geographic-like appearance under the background of GGO, or the distortion of the lung structure and reticular opacities [2]. Crazy-paving pattern is defined as thickened interlobular septa and intralobular lines on the background of ground glass turbidity [2]. Subpleural distribution is defined as the lesion involving the peripheral 1/3 of the lung, while diffuse distribution is defined as continuous involvement without respect to lung segments [1,3].

Lungs are classified into five lung lobes. Left lung is divided into upper and lower lobes by oblique fissure (n = 2), while right lung is divided into upper, middle and lower lobes by horizontal fissure and oblique fissure (n = 3). The number of lobes was recorded based on the presence or absence of COVID-

19 pneumonia in each lobe: 0: no lesion; 1: only one lobe is affected, and so on. If all 5 lobes are affected, the highest number is 5.

We proposed a lung segment-based CT severity score to assess the severity of the COVID-19 pneumonia. Each lung was divided into 10 segments: upper lobe, n = 3; middle / lingulae lobe, n = 2; lower lobe, n = 5) [4-6]. The CT severity score was recorded based on the presence or absence of disease in each segment: 0: no lesion; 1: with lesion, regardless of the degree and range of the lesions. If all 20 segments are affected, the highest score is 20 (**Figure S2**).

Any disagreement was resolved through discussions and consultations.

References

1. Song F, Shi N, Shan F, Zhang Z, Shen J, Lu H, et al. Emerging coronavirus 2019-nCoV pneumonia. Radiology. 2020; 295: 210-7.
2. Hansell DM, Bankier AA, MacMahon H, McLoud TC, Muller NL, Remy J. Fleischner Society: glossary of terms for thoracic imaging. Radiology. 2008;246:697-722.
3. Ooi GC, Khong PL, Müller NL, Yiu WC, Zhou LJ, Ho JC, et al. Severe acute respiratory syndrome: temporal lung changes at thin-section CT in 30 patients. Radiology. 2004;230:836-44.
4. Chae EJ, Seo JB, Jang YM, Krauss B, Lee CW, Lee HJ, et al. Dual-energy CT for assessment of the severity of acute pulmonary embolism: pulmonary perfusion defect score compared with CT angiographic obstruction score and right ventricular/left ventricular diameter ratio. AJR Am J Roentgenol. 2010;194:604-10.
5. Netter FH. Netter's Atlas of Human Anatomy (fifth edition). United States of America, SAUNDERS ELSEVIER.
6. [Internet] Bronchopulmonary segments: annotated CT. Accessed at April 30, 2020. https://radiopaedia.org/cases/bronchopulmonary-segments-annotated-ct-1.

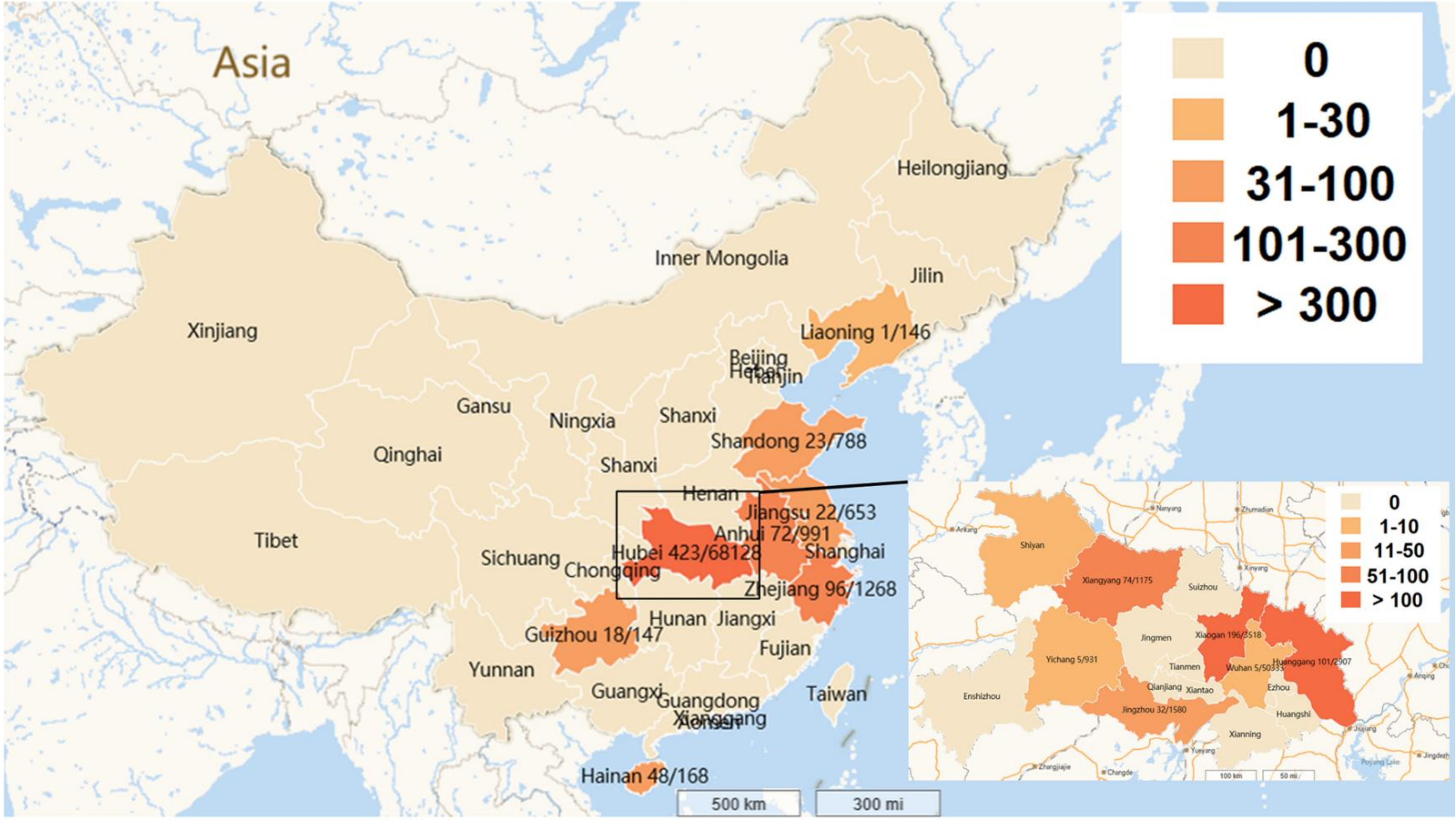

Figure S1: Distribution of COVID-19 patients in this study

This study enrolled 703 confirmed patients from 8 provinces including Hubei (first), Zhejiang (fourth), Anhui (Sixth), Shandong (eighth) and Jiangsu province (ninth), which are the top 10 provinces for the number of confirmed COVID-19 patients as for May 5, 2020. We provide the patients number included in this study (The first number) and overall confirmed patient number (latter number) in each province in China and each administrative city in Hubei included in this study.

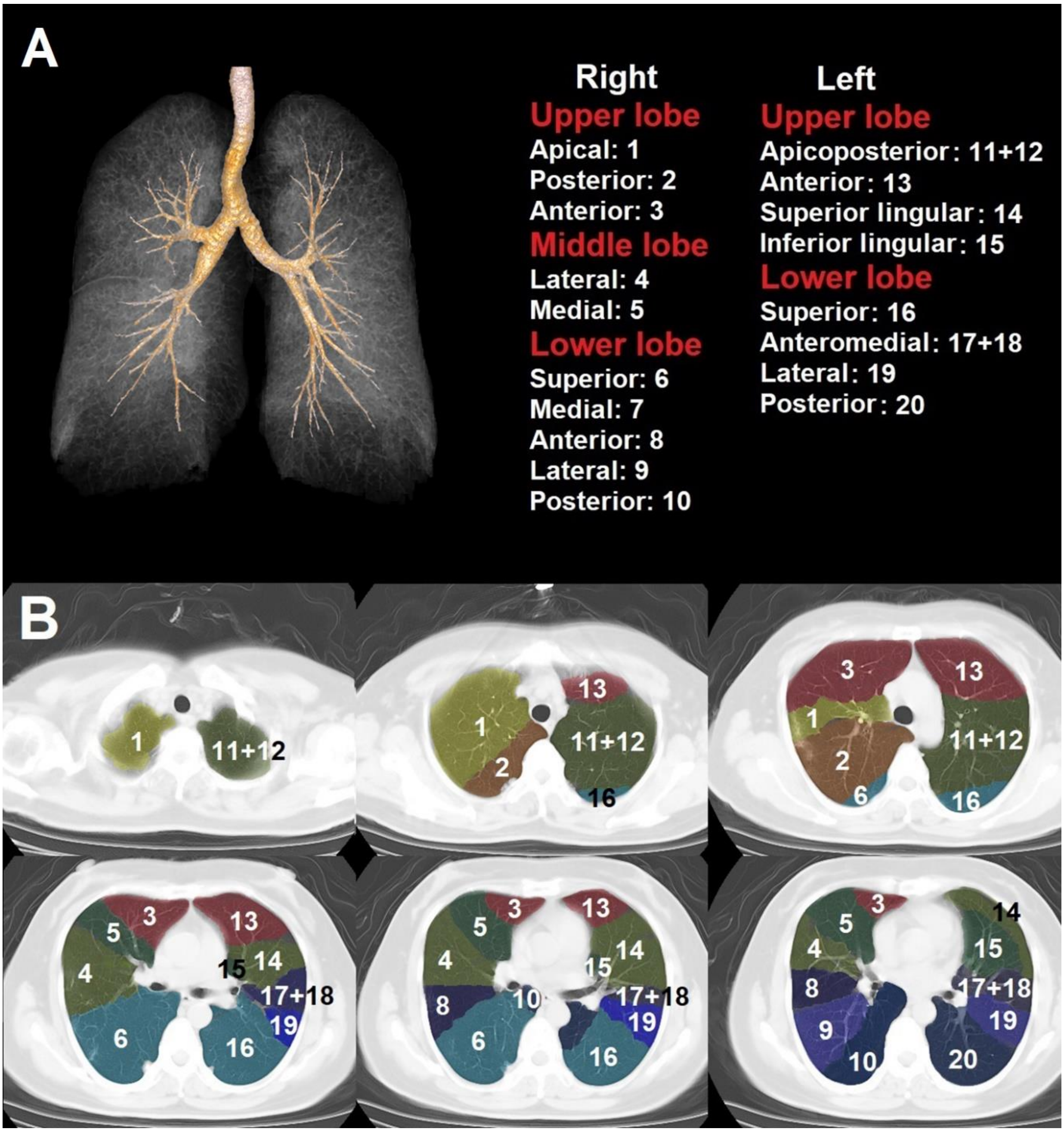

Figure S2: The Division of Lung Segmentation on CT Imaging

Panel A. VR of lung and lung segmentation; Panel B. Division of lung segmentation on cross plane of CT Imaging. As shown, each lung was divided into 10 segments: upper lobe, n = 3; middle / lingulae lobe, n = 2; lower lobe, n = 5).

VR: virtual reality.
